# In search of an ideal template for therapeutic genome editing: A review of current developments for structure optimization

**DOI:** 10.3389/fgeed.2023.1068637

**Published:** 2023-02-22

**Authors:** Alena Shakirova, Timofey Karpov, Yaroslava Komarova, Kirill Lepik

**Affiliations:** ^1^ RM Gorbacheva Research Institute of Pediatric Oncology, Hematology and Transplantation, Pavlov University, Saint Petersburg, Russia; ^2^ Peter the Great St. Petersburg Polytechnic University, Saint Petersburg, Russia

**Keywords:** genome editing, knock-in, donor template, aav, plasmid DNA, dsDNA, ssDNA, ODN

## Abstract

Gene therapy is a fast developing field of medicine with hundreds of ongoing early-stage clinical trials and numerous preclinical studies. Genome editing (GE) now is an increasingly important technology for achieving stable therapeutic effect in gene correction, with hematopoietic cells representing a key target cell population for developing novel treatments for a number of hereditary diseases, infections and cancer. By introducing a double strand break (DSB) in the defined locus of genomic DNA, GE tools allow to knockout the desired gene or to knock-in the therapeutic gene if provided with an appropriate repair template. Currently, the efficiency of methods for GE-mediated knock-in is limited. Significant efforts were focused on improving the parameters and interaction of GE nuclease proteins. However, emerging data suggests that optimal characteristics of repair templates may play an important role in the knock-in mechanisms. While viral vectors with notable example of AAVs as a donor template carrier remain the mainstay in many preclinical trials, non-viral templates, including plasmid and linear dsDNA, long ssDNA templates, single and double-stranded ODNs, represent a promising alternative. Furthermore, tuning of editing conditions for the chosen template as well as its structure, length, sequence optimization, homology arm (HA) modifications may have paramount importance for achieving highly efficient knock-in with favorable safety profile. This review outlines the current developments in optimization of templates for the GE mediated therapeutic gene correction.

## 1 Introduction

Gene therapy is a fast developing field of medicine with hundreds of ongoing early-stage clinical trials and numerous preclinical studies of novel products. On the difficult path of early successes and pitfalls, the retro/lentiviral mediated cell gene therapy translated in a set of breakthrough therapies, such as HSC based treatments for ADA-SCID (Aiuti et al., 2017), metachromatic leukodystrophy (Fumagalli et al., 2022), thalassemia (Locatelli et al., 2022), as well as CAR-T therapy (Leahy et al., 2021; Mikkilineni and Kochenderfer, 2021). Nowadays, the GE extends these therapeutic possibilities by using sequence-specific engineered nucleases mediating controlled and stable gene correction. By introducing a DSB in the defined locus of genomic DNA, these GE tools allow to knockout the desired gene or to knock-in the therapeutic gene if it is accompanied by repair template. The most commonly used types of gene-editing nucleases are zinc finger nuclease (ZFN) (Urnov et al., 2010), transcription activator-like effector nuclease (TALEN) (Joung and Sander, 2013), and clustered regularly interspaced short palindromic repeats-associated nuclease Cas9 (CRISPR/Cas9) (Ran et al., 2013). They all are enzymes, capable of recognizing a specific sequence and producing a DSB in the DNA. Alternatively, single-stranded DNA break made by different types of engineered nickases has also been described (Wu et al., 2014; Rahman et al., 2021)

**FIGURE 1:**
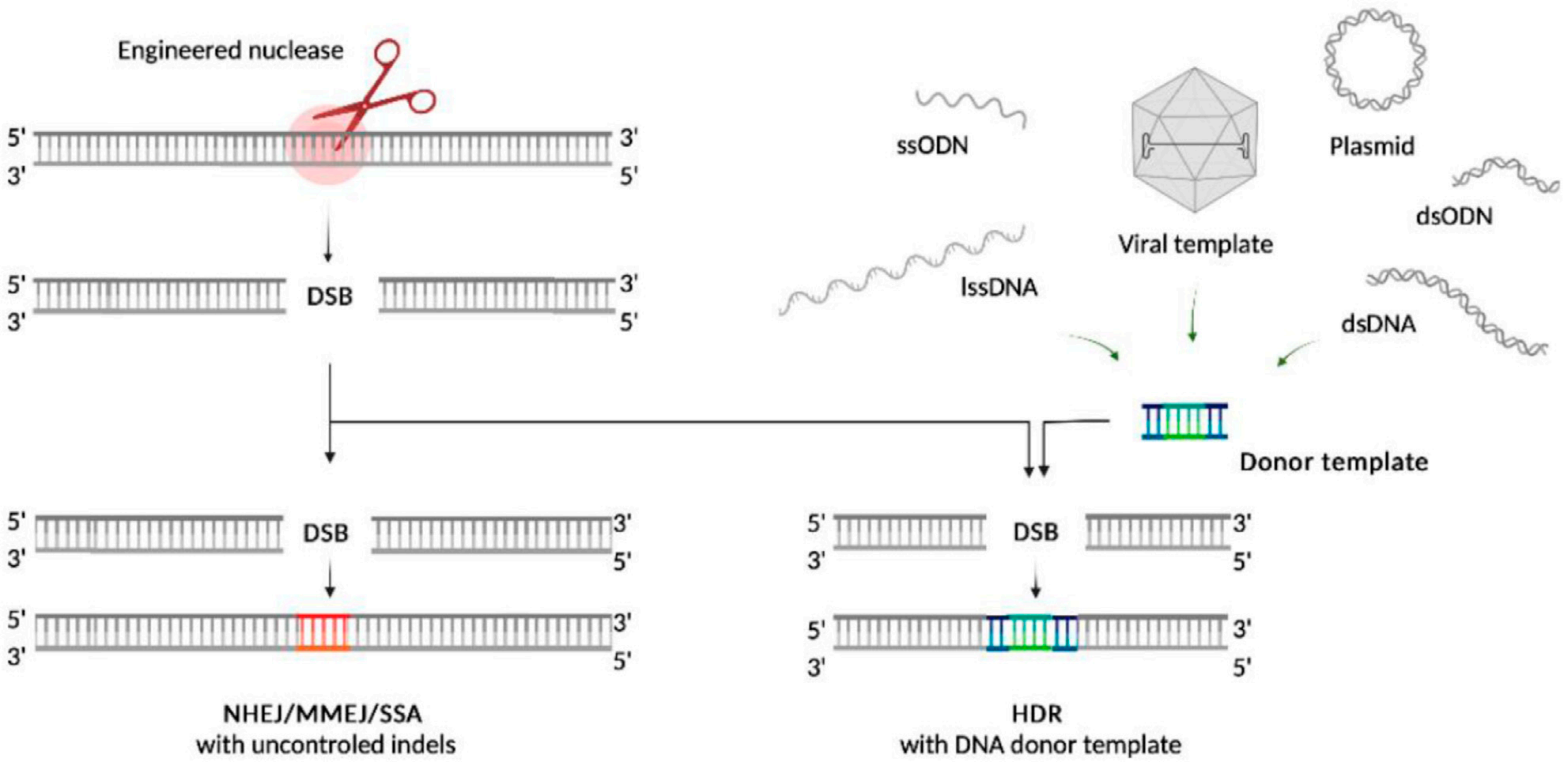
The main DSB repair pathways.

There are two main pathways that repair DNA DSBs: fast re-ligation of the broken DNA ends including non-homologous end joining (NHEJ), microhomology-mediated end joining (MMEJ) and single-strand annealing (SSA) mechanisms, or the homology-directed repair (HDR) using donor templates (Figure 1). Many factors influence the repair pathway choice (Yeh et al., 2019). Fast re-ligation is a default form of repair in human cells, which is active throughout the cell cycle except for mitosis. It rejoins DSBs more quickly than the HDR, permitting uncontrolled small genetic aberrations (Anzalone et al., 2020). Conversely, HDR enables the accurate and controlled sequence repair, and plays important role for gene therapy development. At the same time, HDR is restricted to S/G2 phases of cell cycle (Paulsen et al., 2017) and takes place in the presence of orientation-specific DNA donor templates with appropriate homology regions. Such a limitation makes HDR less efficient, especially in human long- or non-proliferating cell populations, critical for the development of HDR-based technologies for clinical application. The capability of the template mediated HDR to introduce controlled point mutations has also clinical significance, especially for hematopoietic stem/progenitor cells (HSPCs) based therapy (Azhagiri et al., 2021). In this way, increased efficiency of HDR and targeted knock-in is an area of particular interest.

To date, several strategies to avoid limitations and improve HDR-mediated gene editing in human cells have been reported, i.e., suppression of the NHEJ pathway (Chu et al., 2015) or activation of HDR promoting factors (Jayavaradhan et al., 2019), modulation of the cell cycle (Wienert et al., 2020) and modifications of the engineered nucleases (Charpentier et al., 2018). Furthermore, the tandem paired nicking (TPN) method with nicking design application has been developed to address the problem of DSB-induced p53 signaling activation (Benitez et al., 2020) without significant loss of HDR efficiency (Rahman et al., 2021). Even though all these methods can increase the knock-in rate, efficacy and safety sufficient for clinical use is still a challenge.

Turning to the main objective of this review, it must be noted that the type of the donor template can also affect the result of GE. For example, the double-stranded (ds) and single-stranded (ss) templates can serve as substrate for homologous recombination (HR) and single-stranded template repair (SSTR) pathways respectively (Yeh et al., 2019). Both repair mechanisms are still not fully understood, but what is known is that they differ in some of the involved molecular actors as well the intracellular immune response state. RAD51 recombinase and its paralogs activate the searching mechanisms for a double-stranded template to promote an integration of exogenous sequences during DNA repair by HR, while the SSTR is a RAD51-independent process. In SSTR, RAD52 mediates annealing of a single-stranded donor template spanning the break site without incorporating it directly (Gallagher and Haber, 2021). Therefore, the type of the template or the design thereof (e.g., homology arm length or 3’- vs. 5’-end orientation) can influence the knock-in mechanism, which makes this a fundamental factor in the development of gene therapy approaches (Ranawakage et al., 2021). The choice of the template type for HDR should also be consistent with the delivery system and the type of the engineered nuclease used depending on the clinical tasks (Wan et al., 2019). According to the available evidence, the donor template’s classification has not been established in full. Conventionally they could be simply classified by: 1) cargo capacity (short or long), 2) nature (viral or non-viral) and 3) structure (single- or double-stranded) and some other factors (Table 1).

**TABLE 1:** The donor template’s classification.

	Insert size	Single-stranded templates	Double-stranded templates
Non-viral	**Long**	lssDNA	Plasmids, linear dsDNA
	**Short**	ssODN	dsODN
Viral	**Long\Short**	AAV, IDLV	AdV
Hybrid\unclassifiable			

Short templates, in particular oligodeoxynucleotides (ODNs), are comparatively limited in cargo capacity and usually do not exceed 200 bp. As for the long templates, they might be viral and non-viral, while the long viral templates are strictly limited in length due to their native genome cargo capacity in contrast with the non-viral ones (e.g., plasmids and long linear DNAs), which in their turn are limited only by the plasmid carrying capacity and manufacturing capabilities. Viral donor templates are the modified nucleic acids commonly derived from lentiviruses or adeno-associated viruses with viral particles representing a delivery system of their own (Chen and Gonçalves, 2016), while the non-viral donor templates are the nucleic acids synthesized as single- or double-stranded molecules, circle or linear long DNAs or ODNs. The template structure directly affects the choice of DSB repair pathway as well as the HDR rate. Pros and cons of each template’s length, nature or structure and the ways to avoid complications are worth considering for genome-editing mediated therapeutic gene correction and are discussed below.

## 2 Viral donor templates for HDR

Viral vectors are by far the most demanded in clinical applications of gene therapy and genome editing. From the great variety of viral vectors, it were mainly lentiviral vectors, integrase defective lentiviral vectors (IDLVs), and adeno-associated virus (AAV) used as donor templates and donor template delivery systems. Since only IDLVs and AAVs have recorded a therapeutically relevant knock-in efficiency in primary human cells of hematopoietic lineage, below we shall elaborate on these particular vector types.

### 2.1 IDLV donors

Integrase-deficient lentiviral vectors are positive-sense single-stranded RNA vectors, with potential cargo delivery of up to 10 kb (Banasik and McCray, 2010). IDLVs are the derivates of integrated lentiviral vectors (LVs) with minimized risk of insertional mutagenesis (Dong and Kantor, 2021). Recombinant IDLVs lack all the viral open reading frames (ORFs) but retain several critical non-coding elements (Rittiner et al., 2020). Non-pleiotropic (class II) point mutations within the int gene encoding IN enzyme reach the complete abolishing of the IDLV’s integration process (Dong and Kantor, 2021). Such point mutations result in episomal persistence of IDLV genome, which is transient and dissipates during cell divisions making such system biologically safe and suitable for donor template delivery.

Lentiviruses are capable of efficient nuclear import across the intact nuclear membrane, which is essential for gene therapy applications. The homology template delivered as IDLV vectors can mediate base editing as well as targeted correction of gene sequence or large transgene knock-in into the intended target site (Genovese et al., 2014). IDLVs have been utilized for gene targeting of the *IL2RG* along with the GFP cassette into the *IL2RG* locus in murine and human HSPCs (Genovese et al., 2014) and for site-specific correction of the sickle mutation in *HBB* (Hoban et al., 2015; 2016)

Until now, when working with human primary T-cell and HSPCs, the knock-in efficiency of the delivered donor templates using IDLV did not exceed 20% (Table 2), which may be due to the high expression of antiviral factors by this cell types and difficult transduction (Karuppusamy et al., 2022). In the clinically significant early human HSPCs population, the insertion efficiency of the repair template delivered as IDLV reached 12% after the transfection protocol optimization with CsH, GSE56 и E4orf6/7 combination (Ferrari et al., 2022). The repressive chromatin structure formed around the IDLV episomal genomes can mediate the low accessibility of IDLV-delivered donor template for DSB repairing (Dong and Kantor, 2021). The use of small molecules that stimulate the expansion of early hematopoietic precursors in culture, in particular, Stemregenin 1 (SR1), and extended preactivation of the cells before transduction contributed to the increased efficiency of knockout (Genovese et al., 2014). These details were successfully applied later in the protocols for AAV donor templates delivery, which will be discussed in the next section.

**TABLE 2:** Efficiency of the *in vitro* molecularly verified knock-in of long repair template coding cassettes delivered by viral vectors: literature data.

Donor type	Donor length	HA length (left/right)	Cell type	Engineered nuclease	Genetic locus	% knock-in rate *in vitro*	References
rAAV6	No data	No data	NK cells	CRISPR/Cas9 (RNP)	*CD38*	57%–85%	Clara et al. (2022)
rAAV6	2.1 kb	0.4 kb/0.4 kb	HSPCs	CRISPR/Cas9 (mRNA)	*CYBB*	40%–80%	De Ravin et al. (2021)
rAAV6	Full AAV6 genome	0.6 kb/0.6 kb	NK cells	CRISPR/Cas9 (RNP)	*AAVS1*	mean 60% (up to 78%)	Kararoudi (2021)
rAAV6	No data	0.801 kb/0.84 kb	CD34 + 133 + 90+ HSCs	CRISPR/Cas9 (RNP)	*AAVS1*	70%	Ferrari et al. (2020)
					*IL2RG*	50%	
					*CD40L*	35%	
rAAV6	4.4 kb	0.6–1.3 kb/0.6–1.3 kb	T-cell	MegaTAL	*CCR5*	9%–60%	Sather et al. (2015)
rAAV6	No data	0.4 kb/0.4 kb	CD34^+^ HSCs	ZFN mRNA	*AAVS1*	Up to 58%	De Ravin et al. (2016)
rAAV6	3.0 kb	0.722 kb/0.758 kb	CD34^+^ HSPCs	CRISPR/Cas9 (RNP)	*WAS*	52.1% ± 10.9%	Rai et al. (2020)
rAAV6	4.4 kb	0.54 kb/0.42 kb	iPSCs	CRISPR/Cas9 (RNP)	*HBB*	51%	Martin et al. (2019)
rAAV6 (single donor)	3.3 kb	0.4 kb/0.4 kb	T-cell	CRISPR/Cas9 (RNP)	*CCR5*	46%	Bak and Porteus (2017)
			CD34^+^ HSC			19%	
rAAV6	Full AAV6 genome	0.95 kb/0.95 kb	T-cell	CRISPR/Cas9 (mRNA)	*TRAC*	45.6%	Eyquem et al. (2017)
rAAV6	codon diverged IL2RG cDNA	0.4 kb/0.4 kb	CD34^+^ HSPCs	CRISPR/Cas9 (RNP)	*IL2RG*	23.2%–45%	Pavel-Dinu et al. (2019)
rAAV6	3.5 kb	0.476 kb/1.428 kb	T-cell	ZFN mRNA	*AAVS1* and *CCR5*	>40%	Wang et al. (2016)
rAAV6	3.095 kb	0.54 kb/0.42 kb	CD34^+^ HSPCs	CRISPR/Cas9 (RNP)	*HBB*	40%	Charlesworth et al. (2018)
rAAV6	3.9 kb	0.985 kb/0.763	T-cell	TRC1-2 mRNA	*TRAC*	38%	MacLeod et al. (2017)
rAAV6	1.1 kb homology region to HBB		CD34^+^ cells	ZFN mRNA	*HBB*	35%	Romero et al. (2019)
rAAV	4.2–4.5 kb	1.0 kb/1.0 kb	T-cell	TALEN	*CD40LG*	20.9–31.7%	Hubbard et al. (2016)
rAAV6	1.56 kb	0.75 kb/0.75 kb	HSPCs	CRISPR/Cas9 (RNP)	*ELANE*	30%	Tran et al. (2020)
rAAV6	2.4 kb sequence homology to HBB with the SNPs		CD34^+^ HSPCs	CRISPR/Cas9(RNP/mRNA)	*HBB*	11%–29%/15%	Dever et al. (2016)
rAAV6	No data	0.801 kb/0.84 kb	CD34^+^ HSPCs	ZFN mRNA	*CCR5*	17%	Wang et al. (2016)
					*AAVS1*	26%	
rAAV6	2.5 kb	0.425 kb/0.425 kb	HSPCs	CRISPR/Cas9 (RNP)	*PKLR*	25.2% ± 7.2%	Fañanas-Baquero et al. (2021)
rAAV6	1.1 kb	0.52 kb/0.52 kb	HSPCs	CRISPR/Cas9 mRNA	*HBB*	23.5% ± 8.3%	Lomova et al. (2019)
rAAV6	2.3 kb	0.162 kb/0.405 kb	HSPCs	CRISPR/Cas9 (RNP)	*CD40LG*	20.8%	Kuo et al. (2018)
IDLV	1.1 kb + HA	0.4 kb	HSPCs	CRISPR/Cas9 mRNA	*HBB*	20%	Hoban et al. (2016)
IDLV	1.1 kb + HA	0.4 kb	HSPCs	ZFN mRNA	*HBB*	18.4%	Hoban et al. (2015)
rAAV6	2.3 kb	0.162 kb/0.405 kb	HSPCs	CRISPR/Cas9 mRNA	*CD40LG*	16.2%	Kuo et al. (2018)
rAAV6	4.4 kb	0.6–1.3 kb/0.6–1.3 kb	CD34^+^ HSCs	MegaTAL	*CCR5*	14%	Sather et al. (2015)
rAAV6	2.3 kb	0.162 kb/0.405 kb	HSPCs	TALEN	*CD40LG*	13.2%	Kuo et al. (2018)
rAAV1	1.946 kb	No data	human fibroblasts	CRISPR/Cas9	*GFP*	11%	Gaj et al. (2017)
rAAV6	GFP + HA	0.4 kb/0.4 kb	B cells	CRISPR/Cas9 (RNP)	*PRDM1*	10%	Hung et al. (2018)
rAAV6 (two donors)	6.5 kb	0.4 kb/0.4 kb	T-cell	CRISPR/Cas9 (RNP)	*CCR5*	9.8%	Bak and Porteus (2017)
			CD34^+^ HSPCs			9.1%	
IDLV	GFP cassette driven by the phosphoglycerate kinase promoter (PGK) or cDNA comprising exon 5 to 8 of IL2RG together with the GFP cassette		CB CD34^+^ cells	ZFN delivered by adenoviral vector (Ad5/35)	*AAVS1 IL2RG*	5%	Genovese et al. (2014)
IDLV	3.4 kb	0.474 kb/1.404 kb	T-cell	ZFN mRNA	*CCR5*	5%	Lombardo et al. (2011)
	3.2 kb	0.801 kb/0.84 kb			*AAVS1*		
IDLV	1.1 kb homology region to HBB		CD34^+^ cells	ZFN mRNA	*HBB*	2%–5%	Romero et al. (2019)
IDLV	4.4 kb	1.3 kb/1.3 kb	T-cell	MegaTAL	*CCR5*	0.5%	(Sather et al., 2015)

Names of genes knocked out in the reference are in italics.

### 2.2 AAV donors

Viral vectors derived from non-pathogenic single-stranded DNA virus and adeno-associated virus are being widely developed for gene therapy as donor template carriers for primary human hematopoietic stem cells (Gaj et al., 2017; Bernaud et al., 2018). They are attractive for inserting long DNA sequences into a genome for three main reasons. Firstly, AAVs can transduce both the dividing and the non-dividing cells so they can serve as suitable carriers for the delivery of long repair templates (Bijlani et al., 2022). Secondly, in addition to their capacity to safely mediate gene delivery, adeno-associated virus vectors are able to stimulate gene targeting *via* homologous recombination using the Rad51/Rad54 pathway even in the absence of a nuclease-induced DSB (Vasileva, 2006). And thirdly, after the infection, the viral capsid endocytosed, transported to the nucleus and enter the nuclear pore complex, where the ssDNA genome is released from the capsid (Junod et al., 2021). Such mechanism is important for intact delivery of donor template because cell transduction efficiency only depends on efficiency of vector internalization, including nuclear delivery and efficiency of uncoating in the nucleus. After that, AAV genome continues to exist as a single-stranded DNA donor template or it can be converted into a circular double-stranded DNA molecule known as episome (Bernaud et al., 2018; Maes et al., 2019). In the presence of homology domains to the desired insertion locus, HDR is preferable from single-stranded forms of the AAV genome. In the absence of homology arms, the insertion into the DSB locus can take place from the episomal form of the virus genome involving the NHEJ mechanism (Bijlani et al., 2022). In this way, the AAVs’ increased targeted integration was originally attributed to the increased availability of vector genomes in the nucleus, increased stability due to the structured ITRs, and their potential to participate in HR. Such properties of AAVs have formed the basis for their domination as donor vectors both *in vitro* and *in vivo* (Table 2) (Sather et al., 2015; Yang et al., 2016; Yin et al., 2016; Pattabhi et al., 2019; Lattanzi et al., 2021; Wilkinson et al., 2021). AAV donors can mediate base editing as well as targeted correction of gene sequence or large transgene knock-in into the intended target site (Wang et al., 2016; Smith K. A. et al., 2018; Lomova et al., 2019; Tran et al., 2020).

The application of GE using AAV donor vectors are limited to DNA packaging capacity of 4.5 kb. Since the homology arms required for efficient HR add at minimum 2 × 0.3–0.4 kb to the vector (Hendel et al., 2014; Kararoudi, 2021), maximum 3.7–3.9 kb is left for desired repairing template. There are more than 300 disease genes with coding sequences exceeding the AAV genome capacity, which cannot be treated by AAV-mediated gene replacement. The genes accountable for Duchenne Muscular Dystrophy (dystrophin: 11 kb), hemophilia A (Factor VIII: 7 kb), and Cystic Fibrosis (CFTR: 4.4 kb) are among them.

Using AAVs vectors for various primary human cells has demonstrated knock-in efficiency from 9 up to 85% (Table 2), which depends on the nuclease chosen, cell type, transgene and homology arms lengths, targeted locus, and other factors. AAV6 capsid variant was shown to have the highest efficiency in donor delivery to a wide variety of cells of hematopoietic lineage, including HSPCs, B cells, T-cell and NK (Dudek and Porteus, 2021; Rogers et al., 2021) as well as for mesenchymal stromal cells (MSCs) (Srifa et al., 2020). Without additional modifications and enrichment of gene-targeted cells the highest knock-in rates were achieved for NK cells possibly due to the long cell expansion phase (Kararoudi, 2021) in AAVS1 locus (De Ravin et al., 2016; Ferrari et al., 2020). That can be explained by the fact that this is the preferred site for integration by wild-type AAV genomes in absence of helper virus infection (Bijlani et al., 2022). Among the primary human cells of hematopoietic lineage, quiescent primary human HSPCs, especially the long-term repopulating HSCs (LT-HSCs), are the most difficult for HDR. This may be caused by the low fraction of LT-HSCs in S/G2 with characteristically poor expression and activity of HDR machinery as well as delayed transit through the G1/S checkpoint (Ferrari et al., 2020). According to the literature data, the stimulation of cell’s proliferation prior to transfection/transduction was an important additional factor for achieving therapeutically relevant knock-in efficiency. Such activation can be induced during the prolonged culture phase before transduction, which can take from 1 to 2 days for HSPCs and T-cell to 7–14 days for NK cells (Eyquem et al., 2017; De Ravin et al., 2021; Kararoudi, 2021; Clara et al., 2022) or in combination with small molecule expansion stimulators for HSPCs like SR1, UM171 (Ferrari et al., 2020; Lattanzi et al., 2021). The choice of the nuclease and the form of its delivery is also important (Sather et al., 2015; Eyquem et al., 2017; Martin et al., 2019; De Ravin et al., 2021).

The possible strategies for increasing the knock-in efficiency of templates delivered by AAV vectors, may suggest a) donor template structure modifications, b) modification of the profile of proteins presented in the cell that serve DSB repair processes depending on the cell cycle phase, c) conditions of cell culturing and transduction of primary human cells, including donor template concentration; d) genomic target site optimization and nucleases modifications, and e) searching for new AAV capsid proteins mutations that increase the efficiency of the primary cells transduction. In relation to the last two points, little has been published so far.

In contrast to IDLV vectors (Cortijo-Gutiérrez et al., 2021), the possibilities of introducing direct modifications to the AAV donor template for knock-in efficiency stimulation are limited due to the rigid structure parameters of AAV’s capsid and genome. Traditionally, it is recommended for homology arms to not exceed 400 bp (Sather et al., 2015). However, in some studies it was suggested that extension of homology arms up to 1.3 kb can lead to a significant increase in the knock-in efficiency (Sather et al., 2015). There are also conflicting findings that increasing homology arm lengths from 400 bp to 1.0 kb did not lead to higher levels of HDR (Hung et al., 2018). Some reports claimed that using asymmetric homology arms appeared to be more efficient for editing of certain genes (Wang et al., 2016; MacLeod et al., 2017). A recent publication describes an attempt to adapt homology arms in a AAV6-delivered donor template to the proposed mechanism of DSB’s ends repair, depending on the stage of the cell cycle, and yielding positive results in inserting target sequences into primary human CD34^+^ HSPCs (Gray et al., 2022). Since the question about the optimal length of homology arms remains open, transfection of cells by a mixture of such templates (Gray et al., 2022) could be an effective solution to increase the knock-in efficiency, and could be verified in the course of further studies.

The timing of the AAV donor template delivery relatively to electroporation remains controversial, especially for HSPCs (De Ravin et al., 2016; Charlesworth et al., 2018; Martin et al., 2019). Apparently due to the fact that the donor-delivering AAV is replication-incompetent and diluted upon mitosis, and also because the donor-dependent HDR-directed targeted addition takes place preferentially in phase S/G2 with the highest concentrations of donor template optimal at the cutting time, such protocol detail is critical and nuclease specific. Some studies suggest that additional role in knock-in efficiency may be played by cell cycle synchronization at cell cycle phases G2/M or S/G2 with nocodazole or selective inhibitor of CDK1, RO-3306 (“RO”) respectively (Lomova et al., 2019). However, it should be noted that nocodazole has been already used in the context of lentiviral transduction of HSCs and had a profound effect on the cell quality (Papanikolaou et al., 2015). In addition to the established known effects which these molecules have in activating the HDR mechanisms, stabilizing the concentration of the delivered template at the desired phase of the cell cycle is one of the mechanisms of their action.

Culturing at low cell density (1 × 10^5^) (Charlesworth et al., 2018) or at low volume of media (Rogers et al., 2021) are aimed to increase the concentration of donor template molecules. Application of high MOI is another common strategy to enhance the efficiency of gene insertion in primary human cells mainly through increased efficiency of transduction and donor template concentration (Ling et al., 2016; Allen et al., 2022). Usually MOI has the range of 5 × 10^4^–1.5 × 10^5^ (Sather et al., 2015; Gaj et al., 2017; Smith L. J. et al., 2018; Martin et al., 2019; Clara et al., 2022), but higher numbers, e.g., from 3 × 10^5^ to 1 × 10^6^ are not uncommon either (De Ravin et al., 2016; Kararoudi, 2021), which is often associated with toxicity to primary human cells (Brown et al., 2017). In some studies, the clinically relevant knock-in efficiency rates in HSPCs were achieved at 0,5 × 10^4^ vector genomes per cell (Wilkinson et al., 2021). To summarize this paragraph, one should keep in mind the possible differences in the methodologies used for in calculation of MOI in different laboratories. Production of clinical-grade rAAV6 is costly and labor-intensive, and the usage of 1 × 10^5^ and higher vector genomes/cell must be justified in every single case.

Several other possible strategies for increasing the efficiency of knock-in, not directly related to the optimization of the structure/concentration of the donor template were also proposed. One strategy is the inhibition of the key components of the NHEJ repair pathway such as DNA ligase IV by Scr7 (Lomova et al., 2019) and adding other small molecules to enhance the HDR mediated GE (for review in HSPCs see Salisbury-Ruf and Larochelle (2021)). Alternatively, the inhibitiion of +1/–1 bp NHEJ events by M3814 and chromatin decondensating by Trichostatin A had an effect with 3-fold increase in HDR efficiency (Fu et al., 2021). Transient inhibition of p53-binding protein ^1^ (53BP1) was reported to increase (2.3-fold) long-term homology-directed repair in quiescent CD34^+^ hematopoietic cells (De Ravin et al., 2021). High efficiency values of gene insertion in the hard-to-edit LT-HSCs population, up to 40%–50%, have recently been reported using viral delivery systems for long therapeutic cassettes based on AAV6 (Ferrari et al., 2020). As for genomic target site optimizations, the transcriptional activity of targeted genome regions during AAV-mediated delivery of donor templates can affect the HR level (de Alencastro et al., 2021; Spector et al., 2021).

The main perspective in the development of virus-mediated delivery of donor templates will be in the search for small molecules specific to a narrow target range in the defined cells populations with the minimized number of side effects and approved to clinical application (Ferrari et al., 2020; 2022). Additionally, searching of new AAV capsid variants (Ling et al., 2016; van Lieshout et al., 2018) and transduction conditions (Rogers et al., 2021) for primary human cells transfection, or even new engineered nucleases mutants with increased activity at HDR-enriched cell cycle phases (Lomova et al., 2019) can be also important. Taking into account the need to address AAV’s safety issues (Bolt et al., 2021; Charlesworth et al., 2022), the recently identified advantages of the IDLV donor template delivery platform in terms of reduced toxicity associated with reduced intracellular DNA loading will make it preferable for those clinical applications where HDR efficiency values sufficient for functional gene correction are required (Ferrari et al., 2022). Undoubtedly, manufacturing costs and the recent adverse events when using AAVs^1^ should direct the particular interest of researchers to non-viral donor templates and to the development of virus-like particles for donor template delivery in gene therapy applications (Gutierrez-Guerrero et al., 2021). The substitution of viral delivery systems with mimicry of the structures of the viral genome as part of the delivered donor templates can also be considered for development in the near future.

## 3 Non-viral donor templates for HDR

### 3.1 Plasmid donors

The limitations of viral donor templates encourage exploratory research into non-viral precision gene therapy. Circular plasmid DNA, which unlike linear dsDNA and ssDNA, requires minimum protocol steps for synthesis and purification, comes to the fore with large-scale GMP-grade plasmid manufacturing commercially available to date (Jing et al., 2021). NHEJ and HDR both can utilize plasmid donor template for targeted knock-in under respectively optimized donor structure (Yoshimi et al., 2021)In the first case, without homology between the donor and the target, the knock-in efficiency usually did not exceed 1% in model human cells (Bachu et al., 2015; He et al., 2016).

Later on, obligate ligation-gated recombination (ObLiGaRe) and homology-independent targeted integration (HITI) technologies have dramatically improved the situation, by up to 2%–30% (Maresca et al., 2013; Suzuki et al., 2016). Both technologies assume the presence of nuclease target sites in the donor plasmid and are adapted for FokI-based and Cas9 nucleases, respectively. Using the homology arms included into the plasmid DNA for the initiation of HDR mechanisms has improved the knock-in efficiency in human model and pluripotent stem cells, helping to achieve up to 90% insertion rate in selected cases (see Prakash et al., 2016 for review). Among notable reports is the PITCh (Precise Integration into Target Chromosome) method, that has successfully demonstrated the applicability of short 5–25 bp homology arms for MMEJ-mediated knock-in in human HEK293T cells with up to 80% efficiency (Yamamoto and Gerbi, 2018). The method is based on PITCh plasmid donors which contained the repair sequence surrounded by microhomology regions (of 20 bp) that mirror the genomic DNA sequence surrounding the cut site, and flanked by two gRNA cut sites (double-cut donor). Approbation of this system with longer homology arms (up to 0.6–2 kb) has lead to the 20% knock-in efficiency in 293T-cells and 5%–13% in human iPSCs cell line (Zhang et al., 2017; Wen et al., 2018). With coordinated paired nicking of the donor and acceptor DNA by RNA-guided nucleases based on CRISPR-Cas9 components, up to 93% integration rate to the AAVS1 “safe harbor” was achieved in model human cell lines and iPSCs (Chen et al., 2017). Concerning the highly efficient mechanism observed in that study, it was considered that Cas9 D10 A nickase induced structural changes in the donor plasmid facilitating high-level accessibility between the nicked genome and donor plasmid and enabling the single-stranded 3′ DNA tails in the genome to anneal to the complementary DNA sequence in the donor plasmid without RAD51-dependent strand invasion (Nakajima et al., 2018). The combined use of CCND1, a cyclin that functions in G1/S transition, and nocodazole, a G2/M phase synchronizer, was reported to double the HDR efficiency to up to 30% in iPSCs in the Zhang and co-authors study (Zhang et al., 2017). According to the Wen and co-authors study, the delivery of Cas9 and reprogramming factor KLF4 in one episomal vector combined with SV40LT has lead to up to 40% knock-in efficiency into PRDM14, CTNNB1, or AAVS1 loci without any selection (Wen et al., 2018).

The available data indicates the achievable knock-in efficacy in primary human T-cell of up to 50% (Table 3). The studies were focused on successful combination of long homology arms and Cas9-cleavage sequences (CCSs) as the parts of HDR template plasmids to insert CAR sequences or reporter genes to the TRAC locus. Small plasmids of ∼2.5 kb in size (pUC57) are commonly used as backbones for primary human cells, and commercially available minicircles and nanoplasmids (<0.5 kb in size) are also used for this purpose (Jing et al., 2021; Oh et al., 2022). Reducing the size of the backbone vector reliably lowers the toxicity of plasmid DNA (Oh et al., 2022), probably because it reduces the overall amount of DNA introduced into the cell at an equal number of molecules of the embedded sequence.

**TABLE 3:** Efficiency of the *in vitro* molecularly verified knock-in of repair template coding cassettes delivered by plasmid donors: literature data.

Donor type	Donor length	HA length (left/right)	Cell type	Engineered nuclease	Genetic locus	% knock-in rate *in vitro*	References
Plasmid DNA	∼2.5 kb	0.5 bp/0.5 bp	T-cell	CRISPR/Cas9 RNP	*TRAC*	24%–57.9%	Oh et al. (2022)
Plasmid DNA	0.844 kb	0.4 bp/0.4 bp	K562	RNA-guided endonuclease	*IL2RG*	33%	Hendel et al. (2014)
Plasmid DNA	∼3.5 kb	0.5 bp/0.5 bp	K562	sgRNA/Cas9 expression plasmid	*BTK*	18.2%	Gray et al. (2022)
Plasmid DNA	0.844 kb	0.4 bp/0.4 bp	K562	TALEN plasmid	*IL2RG*	17%	Hendel et al. (2014)
Plasmid DNA	3.7 kb	No data	T-cell	CRISPR/Cas9 RNP	*TRAC*	16.8%	Jing et al. (2021)

A study ranking the homology arms lengths from 0.1 to 2 kb has demonstrated no significant advantage in the knock-in efficiency for length above 0.5 kb, regardless of the type of backbone used (Oh et al., 2022), and length of 0.5 kb was considered optimal.

Names of genes knocked out in the reference are in italics.

As for the primary human HSC, there are a few reports, which used plasmids as donor templates for targeted gene repair in this cell population mainly because HSCs are thought to be more quiescent and less likely to be actively involved in the optimal stage of cell cycle. The most representative studies were performed on model K562 cells (Table 3). The knock-in efficiency mediated by plasmid DNA donors in K562 cells directly depended on the selected locus and nuclease, nuclease/donor plasmid concentrations, as well as total DSB introduction efficiency (Hendel et al., 2014). For example, HDR plasmid donors carrying *BTK* cDNA from exons 2 to 19 with 500 bp homology arms and Cas9-cleavage sequence have recorded the highest efficiency for DNA integration (18.2%) in comparison to PITCh or HITI donors (6% and 9.7% respectively) (Gray et al., 2022). At the same time, when Hendel’s group tried to insert shorter sequence with 400 bp homology arms into the *IL2RG* locus in K562 cells using TALEN nuclease, the knock-in efficiency was the same (17%) without any additional cut sites, and up to 33% when RNA-guided endonuclease was used (Hendel et al., 2014).

When designing a plasmid-based DNA donor template, one should consider the proximity of the intended HDR insertion site from the nuclease cut site within 50 bp (Merkle et al., 2015). It is also important to disable re-cutting the target DNA after insertion by introduction of appropriate mutations into the nuclease recognition sites or PAM sites in the donor template (Hendel et al., 2014; Gray et al., 2022). Another recommendation is to avoid large plasmid backbone (Jing et al., 2021; Oh et al., 2022). Setting up the experiments on primary human HSCs using advanced forms of nucleases (mRNA or RNP) with combination of different templates (Gray et al., 2022), or adapting their structure for involvement of both NHEJ and HDR repair pathways (Yoshimi et al., 2021) along with obligatory use of small-sized plasmid backbones are perspective studies in this field.

### 3.2 ODNs

#### 3.2.1 dsODNs

As it was demonstrated by Wen and co-authors, the dsODN insertion looks like a NHEJ-depended process. The dsODN’s role as a donor template in therapeutic knock-in strategies is currently under discussion, mainly because it carries a risk of random insertion to the DNA breaks, induced not only by nucleases, but by replication, or other stresses, which are constantly occurring throughout the cell cycle. The general use of dsODNs is predominantly associated with tagging methods detecting a nuclease off-target cleavage, such as Genome-wide Unbiased Identification of DSBs Enabled by Sequencing (GUIDE-Seq) (Tsai et al., 2015) and its analogues (Tang et al., 2018; Miller et al., 2019; Fan et al., 2022; Quan et al., 2022). In addition, asymmetric semi-dsODNs found an application in introducing the epitope tags to study protein functions (Fang and Shinkai, 2021). Available literature suggests that the precise dsODN insertion efficiency ranges from 4.4% to 52% for human cells, depending on targeted locus, cell type, and dsODN dosage (Table 4).

**TABLE 4:** Efficiency of the *in vitro* molecularly verified target knock-in of repair template coding cassettes delivered by dsODN donors: literature data.

Donor type	Donor length	HA length (left/right)	Cell type	Engineered nuclease	Genetic locus	% knock-in rate	References
dsODN	28–34 bp	No data	T-cell, HSPCs, iPSCs	CRISPR/Cas9 RNP	*EEF2, AAVS1, BCL11A*	4.4%–52%	Fu et al. (2021)
dsODN	34 bp	No data	U2OS, HEK293	CRISPR RNA-guided nuclease	*VEGFA* (1–3 sites), *EMX1*, *FANCF*	∼20%	Tsai et al. (2015)
dsODN	20–200 bp	20–200	HAP1	AsCas12a RNP	*EGFP*	10%	Zhao et al. (2022)
dsODN	34–100 bp	No data	HSPCs, HEK293, HUDEP-2	CRISPR/Cas9 RNP	*BCL11A*	∼6%	Papasavva et al. (2022)

Names of genes knocked out in the reference are in italics.

As for the dsODN structure optimization, it is considered that dsDNA cytotoxity is a DNA length-dependent effect due to the activation of cytosolic DNA-sensing pathways (Yu and Liu, 2021). However, reducing the dsODN’s length does not seem to increase insertion frequency (Wen et al., 2021) and does not enhance efficiency of genome editing. Moreover, addition of phosphorothioate linkages at the ends of the DNA strands helps to stabilize ODNs in cells, ensure nuclease resistance and facilitate ligation reactions, and both 5′ and 3′ end-phosphorylated dsODNs show higher integration frequencies in comparison with the dsODNs with 5′ end protection alone (Tsai et al., 2015; Papasavva et al., 2022).

When using dsODNs as a donor template, one should take into account the low specificity of their insertion and look for the ways to improve this characteristic. For this purpose, a “Ligation-Assisted Homologous Recombination” (LAHR) GE method has been proposed as an option (Zhao et al., 2022). It is based on CRISPR/Cas12a nuclease that forms DSBs with DNA overhangs and the dsDNA donor template containing homologous arms with overhangs complementary to the ends generated by Cas12a at the desired locus. The insertion efficiency of LAHR template with 80 bp homologous arms was 3 times higher (6% knock-in) than that of ssODN template.

#### 3.2.2 ssODNs

SsODNs represent an attractive option as an HDR donor template for small controlled mutations or insertions. Main advantages of these templates includes affordable and scalable production, relatively low toxicity and reduced risk of integration by ligation mechanisms. Usually, 200 bp ssODNs containing at least 20 bp homology arms at both ends allow to insert 160 bp into the DSB locus by SSTR mechanism (Richardson et al., 2018).

Today ssODNs are widely used in biotechnology (Saha et al., 2019), cellular and animal models generation (Remy et al., 2017), and in therapeutic applications using Fok1-based nucleases and CRISPR/Cas9 (Chen et al., 2011, 2015; Di Stazio et al., 2021). While ssODN can be co-transfected with nuclease in the form of plasmid DNA (Johnston et al., 2019) and mRNA (Guo et al., 2018), the most promising results were achieved with ssODN paired with Cas9 RNP (Okamoto et al., 2019; Schubert et al., 2021). In a comprehensive study by Schubert et al., knock-in activity mediated by Cas9 RNP with HDR efficiency was tested at 254 genomic loci in Jurkat cells and 239 genomic loci in HAP1 cells, achieving up to 50% HDR rate for selected experimental conditions and genomic loci (Schubert et al., 2021).

The selected reports on the use of ssODN for knock-in in different cell types is presented in Table 5. HSPCs represent an important cell population, clinically relevant for ssODN-mediated repair. The majority of studies performed on HSPCs are focused on optimizing the correction of the *HBB* locus, which offers the hope of developing treatments for TDT and SCD associated with certain point mutations at this locus. DeWitt and co-authors have screened a set of ssODN template designs with asymmetric homology arms in a complex with Cas9 RNP with achievement of up to 33% HDR at the first exon of *HBB* (DeWitt et al., 2016). In a subsequent work by Magis and co-authors, a knock-in level of more than 30% was achieved at HBB in LT-HSCs. The corrected cells were then transplanted to NBSGW mice model. The erythroid differentiation enrichment indicating the survival advantage of corrected alleles *in vivo* was demonstrated (Magis et al., 2022). In a study by Pattabhi and co-authors, the codon-optimized ssODN donor for sickle correction was compared to the respective rAAV6 donor. The efficient repair rates *in vitro* were achieved for both templates (ssODN, 29.6%; rAAV6, 37.5%).

**TABLE 5:** Efficiency of the *in vitro* molecularly verified knock-in of repair template coding cassettes delivered by ssODN donors: literature data.

Donor type	Donor length, bp	Homology arm length (left/right)	Cell type	Engineered nuclease	Genetic locus	% knock-in rate	References
ssODN	Various	Assymetric arms	HEK293	Cas9 RNP	*BFP*	Up to 60%	Richardson et al. (2016)
ssODN	61–121	30–60/30–60	HEK293T	CRISPR/Cas9 plasmid	*ATP7B*	9.5%–58%*	Pöhler et al. (2020) *included selection step
ssODN	106–144	50/50	T-cell HSPCs iPSCs	Cas9 RNP	*AAVS1 EEF2 BCL11A*	12%–57%	Fu et al. (2021)
ssODN	95	40	7 cell lines	ZFNs mRNA	*AAVS1*	7%–57%	Chen et al. (2011)
ssODN	40–100	36–40	HEK293, iPSCs	Cas9 RNP	*EmGFP*	Up to 56%	Liang et al. (2017)
ssODN*	Various	40/40	HAP1 Jurkat	Cas9 RNP	>200 loci	0%–51%	Schubert et al. (2021)
ssODN	20–60	No data	HSPC	ZFN	*HBB*	41%	Hoban et al. (2015)
ssODN	134	50/50	HEK293	Cas9 RNP	*DMD1*	Up to 40%*	Kagita et al. (2021) *included selection step
ssODN	150	60/60, 90/36 36/90	HEK293	CRISPR/Cas9 plasmid	*TNFα*	39%	Di Stazio et al. (2021)
ssODN	101–195	Various	HSPC	Cas9 RNP	*HBB*	6%–33%	(DeWitt et al., 2016)
ssODN	60–90	Various	HEK293T iPSCs	Cas9 RNP	*ZsGreen*	Up to 27%	Okamoto et al. (2019)
ssODN	168	Assymetric arms	HPSC	Cas9 RNP	*HBB*	24.5% ± 7.6%	Pattabhi et al. (2019)
ssODN	168	111/57	HSPC	Cas9 RNP	*HBB*	23.4%	Magis et al. (2022)
ssODN	90	No data	fibroblasts, iPSCs, motor neuron progenitors	CRISPR/Cas9 plasmid	*SMN2*	14%–16%	Valetdinova et al. (2019)
ssODN	181	90/90	IPS	CRISPR/Cas9 plasmid	*HbE*	3%	Wattanapanitch (2021)
ssODN	200	No data	LCL line	CRISPR/Cas9 plasmid	*TBC1D4*	<1.5%	Johnston et al. (2019)

Names of genes knocked out in the reference are in italics.

Importantly, in contrast to higher *in vitro* efficiency of rAAV6 vector, at 12–14 weeks post-transplant into recipient NBSGW mice, a ∼6-fold higher proportion of ssODN-modified cells persisted *in vivo* compared to recipients of rAAV6-modified HPSCs. It can be partially explained by the toxicity of viral vectors impairing HSC engraftment (Romero et al., 2019; Lattanzi et al., 2021). Hoban et al. guessed that longer ssODN strands with prolonged homology arms were optimal for achieving a high gene correction level, inferring the homology arm reduction inefficient. Using longer reverse-strand templates with introduction of silent mutation sites to block a nuclease recleavage allowed them to increase the knock-in from 15% up to 40% (Table 5) (Hoban et al., 2015).

It should be noted that as the ssODN represents a robust platform for screening of different structural modifications and experimental conditions, the results of the cited works cannot be easily presented in concise manner and one should address the original articles for detailed analysis of the study results. The majority of the basic parameters, such as the choice of targeting (T, ODN complementary to the CRISPR–Cas9 gRNA) or non-targeting (NT) strand, length, homology arms size and symmetry, orientation, addition of mutations preventing target re-cut, sequence optimizations, nuclear localization

Sequences as well as chemical modifications was tested in reported studies with only few general considerations that could be drawed from them. The preference for utilizing a donor oligo with sequences either complementary or non-complementary to gRNA may influence the outcomes of genome editing, as T strand binding to the Cas9 may reduce overall editing efficiency. However, the results of the studies addressing this question regarding the HDR rate varied with no universal strand preference concluded to date. Schubert and co-authors in the systematical manner assessed the knock-in activity mediated by Cas9 RNP/ssODN templates at 254 and 239 genomic loci in Jurkat cells and HAP1 cells respectively (Schubert et al., 2021). Phosphorothioate-modified templates contained 40 bp HA and an insert of a six base EcoRI restriction digest recognition site. The HDR rate defined as the precise insertion of the EcoRI sequence at the canonical cut site was quantified with comparisons between templates consisting of either the T strand or the NT strand. Interestingly, no statistical difference was observed in total editing when either the T or NT strand was used in Jurkat cells, while a significant difference in editing efficiency was observed in HAP1 cells (80.2% mean editing for NT strand vs. 67.8% for T strand was used). Consequently, for HAP1 a significantly higher mean HDR rate was observed across all sites when the NT strand was used (20.6%) than the T strand (15.2%). In contrast, a significantly higher mean HDR rate in Jurkat cells was observed for T strand compared to the NT strand (11.3% vs. 7.5%, respectively). The reasons for these cell-type specific differences are currently undeciphered. The same report also included the assessments of the strand preference for the inserts shifted from the Cas9 cut loci and therefore targeted outside optimal distance from DSB (see below). In that case, for PAM-distal insertions, the NT strand donor templates gave ∼6-fold higher mean HDR rate compared to the T strand, while, for PAM-proximal insertions, the T strand gave ∼10-fold higher HDR than the NT strand. Considering these cell type and gRNA specific differences, the testing of particular templates seems to be reasonable in each case.

The analysis of the significance of overall length of ssODN template across the reported studies did not lead to conclusive results among different experimental adaptations (Yang et al., 2013; Richardson et al., 2016). Studies of Cas9 DSB formation kinetics showed that upon binding to its target, Cas9 releases the PAM-distal non-target strand, creating the rationale for designing asymmetric homology arms for sgRNAs that target the sense strand with a long 5′ homology arm and a shorter 3′ annealing arm (DeWitt et al., 2016; Richardson et al., 2016). In a study by DeWitt et al., in a set of tested ssODN donors with Cas9 RNP, the best results of HBB gene correction were achieved using a template with a 111 bp 5′ arm and a 57 bp 3′ arm, proponing longer and asymmetric homology arms to be advantageous for some experimental installments. Such designs were shown to be optimal by other groups (Di Stazio et al., 2021). Other model proposed by Liang et al. implies that after Cas9 nuclease cleaves, both sides of the double-stranded break are recognized by the DNA repair machinery equally, based on observations that both the non-target and target asymmetric HA ssODNs enhanced HDR regardless of the orientation of the Cas9 nuclease compared to a standard symmetrical donor design. The optimal ssODN donor defined having 30–35 base arms 3′- to the insertion/repair cassette and greater than 40 bases on the 5′-end (Liang et al., 2017). However, in a comprehensive study by Okamoto et al. comparing different structures of ssODN templates for single-base substitution in a reporter gene model showed that the optimal ssODN had a 30–35 bp perfectly matched homology arms on both sides (Okamoto et al., 2019). Considering these discrepancies, one standard starting point that could be employed is to design ssODN donor templates with 40-bp homology arms (Schubert et al., 2021).

CRISPR/Cas9 can re-cut the repair site after the successful insert if the protospacer and PAM sequence remained unaltered, lowering HDR efficiency (Schubert et al., 2021). This outcome can be prevented by incorporation of blocking mutations into the donor template, which was shown by several independent groups (Paquet et al., 2016; Okamoto et al., 2019; Schubert et al., 2021). While there are reports showing lack of impact of these modifications in case of disruption of the seed recognition sequence by longer inserts (Liang et al., 2017), the introduction of PAM mutations can be considered an important general aspect for design of ssODN template^2^.

Shubert et al. aimed to define a ruleset for the placement and a number of blocking mutations required to maximize HDR efficiency. HDR ssODN with Cas9 RNP complexes targeting two different genomic loci into HEK293 and K562 cells was tested with assessment of HDR rate. The HDR rate was low (<2%) for all conditions tested in the absence of any blocking mutation, and was greatly increased (8.0%–17.8%) by addition of a blocking mutation in the second or third base of the “NGG” PAM. Blocking mutations placed around the Cas9 cleavage site and near the 3′-end of the guide were also effective, with the impact reduced as the position of the blocking mutation moved PAM-distal. None of four DNA bases were preferred over others. The study showed that two blocking mutations may lead to more robust improvement in HDR efficiency than donor templates containing a single blocking mutation, while incorporating three or four blocking mutations did not further enhance HDR efficiency over the best combination of two (2 PAM or 1 PAM +1 seed). The additional PAM mutations negatively affected the HDR efficiency in some experimental settings, suggesting that there is a limit to the number of additional mutations that should be added to prevent Cas9 re-cleavage. Based on the results of the study the authors^3^ suggested the HDR template tool^2^ that facilitates the placement and number of blocking mutations required (Schubert et al., 2021).

Sequence optimization in regard of bp distance of the insert from the DBS location was also shown to be a factor affecting the ssODN knock-in efficiency. Most reports assessing this parameter show that the insert site should be placed in close proximity to DSB for optimal HDR efficiency (Inui et al., 2015; Liang et al., 2017; Schubert et al., 2021). Unfortunately, the compliance for this requirement is limited in CRISPR based systems as the location of a DSB site is defined by the presence of PAM, which is not the case for Fok1 based nucleases. As an example, Liang and co-authors using ssODNs and Cas9 RNP showed that gRNAs in close proximity (−7, −3, +3, and +5) to the insertion site produced the highest HDR rates (up to 10-fold higher comparing to + -10 bps from DSB site) (Liang et al., 2017). Importantly, as different gRNAs enabling different rate of overall DSB formation, this factor also influences the outcome in regard of HDR rate and should be considered when designing ssODNs\Cas9 editing system. Cas9 nickase RNP complexes targeting both strands in the PAM-out orientation was shown to be more efficient in inserting of templates between flanking nick sites at a location that would be otherwise considered sub-optimal for wild-type Cas9 designs. Alt-CRISPR HDR Design Tool proposed by Schubert et al. also include gRNA selection algorithms for Cas9 to balance the distance from the cut to mutation taking into account the on- and off-target scores of available gRNAs, as well as for Cas9 D10 A nickases, where the gRNA orientation and distance between nick sites is considered. Another predictive model, assisting the design of ssODNs for introduction of point mutations called CUNE (Computational Universal Nucleotide Editor)^3^ considering sequence and homology arms structure was created using machine learning algorithms based on the dataset containing 30 samples (unique HDR targets), from 126 experiments targeting a total of 744 mice (O’Brien et al., 2019).

The use of two sgRNA was shown to increase the HDR rate mediated by ssODNs donors. In a study by Di Stazio et al., the optimized HDR procedure with the use of double sgRNAs, asymmetric ssODN and triple transfection events enabled the increase the *TNFα* gene HDR rate from an undetectable level to 39% in HEK293 cell line model (Di Stazio et al., 2021). In addition, the cell cycle blocking at G2/M phase with nocodazole treatment was reported to increase HDR efficiency (Han et al., 2020). The transcription activity of targeted sequence was also shown to be affecting the knock-in rate, with transcriptionally active sites increasing DSB repair activity (Davis and Maizels, 2014).

As ssODN cannot readily transport trough the nuclear membrane, the addition of NLS-tagged ssODNs represent the strategy for optimization of HDR efficiency. In study by Han et al., 2020 the NLS-tagging of ssODNs enhanced SSTR and small genetic aberrations efficiency by 4-fold compared to the control (Han et al., 2020). Alternatively, the ssODN may be covalently bound to crRNA or even Cas9 enzyme with formation of chimeric RNPs, assisting the nuclear import of the template (Aird et al., 2018; Savić et al., 2019; Ghosh et al., 2022). It has been reported that incorporating chemical modifications such as phosphorothioate linkages may improve HDR when using ssODN donors (Renaud et al., 2016; Liang et al., 2017), while other studies does not confirm the benefit of phosphorothioate modifications of ssODNs regarding the HDR rates (DeWitt et al., 2016).

Despite this controversy, accounting for the relative simplicity and safety of this modification can be one of the basic considerations in ssODN template design. Another route to improve HDR frequency is by using chemical compounds that inhibit key DSB repair enzymes that play a role in the competing NHEJ pathway. Several chemical compounds have been reported to increase HDR mediated by ssODN templates, however this issue is out of the scope of the current review (Ma et al., 2018; Pavel-Dinu et al., 2019; Li et al., 2020).

To sum it up, general recommendations that can be considered when designing the ssODN template are as follows: introduction of 40 bp symmetric homology arms, addition of a blocking mutation in the PAM sequence, localization of insertion in maximal proximity to DSB site. The exact structural modifications and parameters such as targeting T or NT strand may be cell type and genetic locus specific and should be tuned for each experimental setting.

#### 3.2.3 Linear long dsDNA

Linear long double-stranded DNA is the one of the most widely developed forms of donor template for clinical translation (Quadros et al., 2017; Mayo-Muñoz et al., 2018; Roth et al., 2018; Yang et al., 2022). Such templates can repair DSB using HR, MMEJ and SSA mechanisms, depending on the homology arm’s length. They are usually used to insert long fragments (>1 kb) and make it possible to encode several required sequences and obtain biallelic gene insertions as well as multiplex 2x/3x gene modifications (Agudelo et al., 2017; Barylski et al., 2020; Branton et al., 2020; Zhang et al., 2022).

The most studies that used linear long dsDNA as a donor template were carried out using the CRISPR/Cas9 (Ménoret et al., 2015), but applications of other nucleases were also reported (Mino et al., 2009; Jinek et al., 2013; Zhang et al., 2019; Yang et al., 2022). The usage of Cas9 nickase modified to reduce off-target double-strand breaks was also efficient in increasing of the template homologous insertion into the desired locus in some studies with reduced genotoxicity profile (Mali et al., 2013; Ran et al., 2013). A small number of works have been devoted to the application of the linear type dsDNA template on primary human cells. Model cell lines and organisms are the prevailing objects in this field (Gutierrez-Triana et al., 2018). The knock-in efficiency when using dsDNA linear templates reached 5%–70% (Table 6), but still remains relatively inefficient for many cell types (Chen et al., 2011). Efficiency was minimal in nerve cells and melanocytes, but was above 50% in human hematopoietic cell lines, human pronuclear zygotes, and some other model cells.

**TABLE 6:** Efficiency of the *in vitro* molecularly verified knock-in of repair template coding cassettes delivered by long dsDNA donors: literature data.

Donor type	Donor length	Homology arm length (left/right)	Cell type	Engineered nuclease	Genetic locus	% knock-in rate	References
dsDNA	2000	800/800	human ripronuclear zygotes	CRISPR/Cas9	*OCT4*	18%–27%	Yao et al. (2018)
dsDNA	700	50/50	HEK293T, hESC H1, iPSCs WTC G3	CRISPR/Cas9	*GAPDH, CCR5, AAVS1*	42%–65%	Yu et al. (2015)
dsDNA	566	90/90	HEK293T, K562	CRISPR/Cas9	*TOMM20, GAPDH, SEC61B, EMX1*	52%	Ghanta et al. (2021)
dsDNA	750	300/300	Human T-cell	CRISPR/Cas9	*RAB11A*	up to 50%	Roth et al. (2018)
dsDNA		600/600	HEK293T	CRISPR/Cas9	*CTNNB1*	30%	Zhang et al. (2017)
dsDNA-ssDNA	55 (substrate DNA)	36/91	HEK293, K562	CRISPR/Cas9	*BFP, CXCR4, CCR5, EMX1*	16%	Richardson et al. (2016)
dsDNA	334	400–800/400–800	K562	TALENs	*IL2RG*	15%	Hendel et al. (2014)
dsDNA	1500	350/350	Human T-cell	CRISPR/Cas9	*TRAC*	2%–5%	Nguyen et al. (2020)

Names of genes knocked out in the reference are in italics.

According to the literature data, successful HR requires the creation of dsDNA templates with length of homology arms within the range of 0.2–1 kb (Canaj et al., 2019; Zhang et al., 2022). Some studies showed that homology arms with a length of 100–200 bp demonstrate less HDR efficiency than 400–800 bp arms when targeting *IL2RG* locus in K562 cells (Hendel et al., 2014). The difference in knock-in efficiency between templates with homology arms of 400 and 800 bp was insignificant. This fact allows assuming that for the most efficient nuclease-induced HR in human cells, the homology arms at both ends must be approximately 400–800 bp in length (Hendel et al., 2014; Zhang et al., 2022). Jian-Ping Zhang’s team confirmed this hypothesis in HEK293T cells, where the dsDNA template with homologous arms of 200–300 bp showed 0.22% of HDR efficiency, while the template with homology arms of 600–800 bp increased HDR up to 10% (Zhang et al., 2017). Same group reported that the combined use of double cut donors with homology arms of 600 bp with cell cycle regulators (Nocodazole and CCND1) increased knock-in by up to 20%–30% (Zhang et al., 2017).

An important factor that affects the HDR efficiency is the length of dsDNA donor template. It has been found that ultra-long (2 kb) and ultra-short (<250 bp) DNA templates lead to a decrease in HDR efficiency compared to 951 bp dsDNA (DiNapoli et al., 2020). Moreover, the toxicity of linear dsDNA templates is length-dependent (Paludan et al., 2019), especially for primary human cells (Zhao et al., 2006; Hornung and Latz, 2010; Luecke et al., 2017; Roth et al., 2018). The delivery of CRISPR-Cas9 as RNP can partially overcome this problem (Roth et al., 2018; Nguyen et al., 2020). The activation of intracellular dsDNA sensors in response to non-viral donor template delivery should be taken into account (Piras and Kajaste-Rudnitski, 2021) as an important toxicity mechanism. According to some literature data, the temporary inhibition of cytosolic DNA sensors increased the viability and CAR insertion rates after DNA transfection of primary human T-cell (Kath et al., 2022).

Yao and co-authors tested a set of configurations of linearized dsDNA templates with long 800 bp homology arms (Yao et al., 2018). The dsDNA linearized *in vitro* by digestion with two restriction enzymes with 0–2000 bp non-homologous junk sequence adjacent to Has, was compared with the two types of plasmid donors either or not containing sgRNA binding sites next to HAs. The dsDNA donor that did not contain a non-homologous sequence adjacent to the homology arms (“Tild” donor) demonstrated better knock-in efficiency (33%) than either the plasmid donor with sgRNA binding sites (21%) or the plasmid lacking sgRNA binding sites (0%) at the Actb gene in mouse zygotes. OCT4-sgRNA, Cas9 mRNA and the Tild *OCT4* donor were co-introduced into human pronuclear zygotes. The Tild template has demonstrated higher knock-in efficiency than the plasmid HR template (21% vs. 2% for a single blastomere and 71% vs. 11% for a whole embryo.

David N. Nguyen et al., (2020) attempted to integrate the Cas9 nickase with RNP and CTS in the HDR template to enhance nuclear import and knock-in rate. The HDR template was modified to encode 20 bp Cas9 target sequences at the ends of the homology arms. The use of CTS-modified HDR templates in conjunction with dCas9-NLS RNP increased the HDR up to 1.5% compared to 0.6% in control samples. Next, HDR template was synthesized with short CTS (sCTS) 16 bp long and catalytically active Cas9-NLS RNP was used instead of dCas9-NLS RNP. The sCTS provide binding to Cas9 but exclude cutting (Jiang and Doudna, 2017). This approach was reported to increase the insertion efficiency of 1.5 kb dsDNA into the *TRAC* locus of human T-cell (5% vs. 1.5%).

Another proposed approach to improve knock-in efficiency is the use of linear dsDNA along with an asymmetric sgRNA site located at the 3′ end (DiNapoli et al., 2020). The application of such a technology at mitfa gene (b692) using resulted in a 9% phenotypic recovery in the embryos. Christopher D Richardson et al. investigated the interaction of Cas9 with a donor template in order to increase the efficiency of HDR (Richardson et al., 2016). It was demonstrated that Cas9 asymmetrically releases the 3′ end of the cleaved DNA strand, which is not complementary to sgRNA. Based on these data, the Cas9-dsDNA-ssDNA complex was developed, which was reported to achieve the level of HDR up to 16% in human cell lines (HEK293, K562).

One of the limiting factors that reduce the knock-in efficiency of templates delivered as linear dsDNA is their multimerization and non-homologous end joining during reparation. According to the literature data, the chemical protection of the template ends by biotin or carbon spacers (SpC3) prevented multimerization and resulted in knock-in increase up to 9.5% among the survived medaka zygotes (Gutierrez-Triana et al., 2018). Ghanta and co-author’s group on human cells tested the similar approach (Ghanta et al., 2021). The dsDNA donors (566 bp) with 90 bp homology arms were modified at the 5′-terminus with 2′OMe-RNA fused to triethylene glycol of different length. The HDR frequency was increased up to 52% for triethylene glycol compared to 25% when using unmodified dsDNA in model HEK293T cells. Moreover, 2′OMe-RNA/triethylene glycol-modified donors participated in the same number of HDR events.

Lee et al. selected several modifications for dsDNA template ends to increase the gene knock-in (Lee et al., 2019). They included an amine group with a C6 linker, C12 linker to the donor template and conjugated the linker’s amine groups with their *N*-hydroxysuccinimide esters. Additionally, the phosphate linkages were replaced with stable phosphorothioate linkages at the donor template ends. The modified dsDNA template has been tested on GAPDH locus in HCT116 cells. The dsDNA templates modified with phosphorothioate was reported to be efficient with 1.8х increase of knock-in. At the same time, C6 and C12 linkers enhanced knock-in rate to 4x and 3.8x respectively. It is important to note that all 5′-modifications of the template resulted in an increase in the knock-in level, while the 3′-modifications showed no changes or a decreased the gene correction rate.

Summarizing what has been set forth above, the structure of long dsDNA templates is important for efficient knock-in. Variation of homology arms and transgene lengths greatly affects the knock-in efficiency. According to the works described above, templates with 400–800 bp homology arms and approximately 1000 bp in length are the most promising. Also, chemical DNA end protection and the application of cytosolic DNA sensors inhibitors can be proposed as potential strategies to increase the efficiency of dsDNA incorporation into the required loci and preserve the proportion of viable transfected cells.

#### 3.2.4 Long ssDNA

While historically dsDNA was firstly used as synthetic donor templates with subsequent wider adoption, its shortcomings may hamper therapeutic gene editing applications. Such applications include cases of duplication of homology arms or partial incorporation of the dsDNA template as it is more readily inserted by the dominant NHEJ mechanism (Wen et al., 2021) at off-target DSBs or endogenous DSBs, as well as high toxicity and activation of inflammatory response by dsDNA templates which impair cell viability and transfection efficiency (Yu and Liu, 2021). To avoid these undesired consequences, researchers have turned their attention to long single-stranded DNA templates as homology recombination donors. While the most common forms of homology recombination in somatic cells are completely dependent on the action of the Rad51 recombinase (Chen et al., 2008), lssDNA donors are utilized in Rad51-independent SSTR pathway (Gallagher et al., 2020; Gallagher and Haber, 2021). The key advantage of the lssDNA donors in terms of safety is the absence of integration by direct ligation of donors with DSB end by NHEJ and minimal risk of non-specific insertions in loci independent of target homology, translating in higher knock-in specificity (Roth et al., 2018). Moreover, end-joining ligation reactions assemble linear dsDNA molecules into concatemers in eukaryotic cells, limiting the number of individual donor molecules and their ability to diffuse to their DSB target sites, which is not the case for ssDNA.

The ssDNA was also demonstrated to exhibit lower toxicity (Roth et al., 2018), which is especially important for applications involving scarce populations of primary cells, such as HSPCs. Currently long ssDNA donors are mostly need to be generated manually by one of the reported methods, all of which are comparably laborious. Due to this aspect, the data regarding the application of lssDNA donors is limited, especially in primary human cell types (Table 7).

**TABLE 7:** Efficiency of the *in vitro* molecularly verified knock-in of repair template coding cassettes delivered by long ssDNA donors: literature data.

Donor type	Donor length	Homology arm length (left/right)	Cell type	Engineered nuclease	Genetic locus	% knock-in rate	References
lssDNA	Various	300–600 bp	T-cell	Cas9 RNP	*TRAC*	Up to 80%	Shy et al. (2022)
lssDNA	different	different	HEK293T	Cas9 RNP	*CLTA*	20%–40%	Li et al. (2019)
lssDNA	689	339/347	T-cell	Cas9 nickase	*IL2R*	24.9%*	Roth et al. (2018)
lssDNA	3,5 kb	300/300	T-cell	Cas9 RNP	*IL2R*	5%–20%	Lin-Shiao et al. (2022)
lssDNA	700	40/40	HEK293T	Cas9 plasmid	*SP3*	16%	Katayama and Andou (2021)

Names of genes knocked out in the reference are in italics.

The successful use of lssDNA donors with Cas9 RNP’s for generation of gene modified animal strains was demonstrated in a series of reports (Bennett et al., 2021; Zhang et al., 2022). However, the quantitative assessment of the knock-in efficiency in these cases is challenging. Using Cas9 DNA plasmid and lssDNA donors the knock-in of the methylated SP3 promoter was demonstrated by Katayama and Andou with estimated efficiency of about 16% (Katayama and Andou, 2021). Roth et al. reported the successful correction of *IL2RA* c.800delA mutation in T-cell using D10 A Cas9 nickase and long ssDNA donor, demonstrating 24.9% IL2RA expression rate. However, this result should be considered with caution, as addressed frameshift mutation could be corrected both by HDR as well as NHEJ, presumably due to some of the small indels restoring the open reading frame (Roth et al., 2018). In the most comprehensive study to date by the same group, the performance of lssDNA donors harboring different structural modifications was evaluated across a variety of clinically relevant primary cell types including CD4^+^ T-cell, CD8^+^ T-cell, regulatory T-cell, NK cells, B cells, HSPC’s and gamma-delta T-cell with ultra-high HDR efficiencies (>80–90%) in selected optimal conditioned experiments. Importantly, in all evaluated cell types lssDNA templates demonstrated significantly lower toxicity, increased knock-in efficiency, and generated higher absolute knock-in cell yields comparing to dsDNA donors containing the corresponding structural modifications (Shy et al., 2022). Based on the developed protocol, the group created a GMP-compatible method for fully non-viral CAR-T cell manufacturing, demonstrating knock-in efficiencies of 46%–62% and generating yields of >1.5 × 10^9^ CAR + T-cell.

While the number of systematical studies regarding gene correction implying lssDNA is limited, the current data suggests that length, concentration, and addition of CTS hairpins may be important factors influencing the results of editing.

The experiments assessing the role of the template length included comparison of tNGFR (1500 bp), IL2RA-GFP (2267 bp) bearing lssDNA templates of similar structure targeted to a *IL2RA* locus and BCMA-CAR (2923 bp) targeted to TRAC locus with 78.5%, 38%, 39% maximum transgene expression rate, correspondingly, confirming the observations that shorter templates facilitate higher HDR efficiency (Shy et al., 2022).

In a comparative study by Li et al., a GFP (∼700 bp) insert flanked by various sizes HA (36–700 bp) was targeted to *RAB11A* in HEK293T cells with a near-exponential relationship between homology length and knock-in efficiency: longer homology arms were shown to drive higher efficiency, with 95% of maximal efficiency reached using ∼400 bp arms (Li et al., 2019).

As the nuclear transport of DNA template represent one of the key barriers for efficient gene correction, the modification of the template structure by introduction of Cas9 Target Sequences (CTS), allowing the co-electroporated RNPs to bind the templates and facilitate their delivery was proposed (Nguyen et al., 2020). Shy et al., 2022 screened 10 different template CTS designs using short 113–195 bp ssDNA templates (Shy et al., 2022). While the majority of applied ssCTS configurations increased knock-in efficiency, the group considered the design that incorporated CTS sites in annealed complementary oligonucleotides to be optimal in terms of efficiency, toxicity and preparation procedures. Addition of CTS also increased the knock-in rates of lssDNA donors. CTS sites with scrambled gRNA sequence had not increased knock-in efficiency, suggesting specific recognition of the gRNA sequence. Importantly, only the 5′ CTS was shown to be functional in the studied designs which could reflect requirements for RNP binding and orientation, intracellular trafficking, or interference with repair machinery during 3′ annealing of long ssDNA (Shy et al., 2022).

In report by Lin-Shiao and co-authors the similar approach involved ∼3.5 kb multigene lssDNA cassette which contained truncated Cas9 target sequences to increase shuttling into the nucleus, complexed to DNA nanostructures. This approach enabled up to 20% knock-in to *IL2RA* in primary human T-cell (Lin-Shiao et al., 2022).

5′-Modifications may improve potency and efficacy of DNA donors for precision genome editing. Ghanta et al., (2021) reported that addition of RNA: TEG (triethylene glycol) at the 5′-end of a long (800 bp) ssDNA donor significantly boosted HDR in HEK293T model. The frequency of HDR increased with the dose of ssDNA donor, reaching maximal HDR (22.5%) at 6–8 pmol donor amounts.

A panel of small molecule inhibitors reported to enhance knock-in efficiency of lssDNA donors in primary human T-cell including NU7441 and M3814 (DNA-PK inhibitors), the Trichostatin A (HDAC class I/II Inhibitor), XL413 (CDC7 inhibitor), Alt-R HDR Enhancer (an IDT’s proprietary NHEJ inhibitor). At optimal concentrations, M3814 showed the largest effect size (∼49% increase), followed by XL413 (∼46% increase), NU7441 (∼43% increase), Alt-R HDR Enhancer (∼29% increase), and TSA (∼16% increase). By introduction of MT and MTX inhibitor combinations Shy et al. were able to achieve a knock-in rate of >90% in some experiments involving 1.5–2.7 kb lssDNA donors with CTS structural modifications (Shy et al., 2022).

Results of HDR experiments invoving lssDNA show that this type of templates is highly promising for therapeutic GE due to the improved safety profile. The starting points when designing lssDNA templates are symmetrical 400 bp homology arms, introduction of CTS sequences and sequence optimization for overall repair template length reduction.

## 4 Conclusion

The analysis of the current research data shows that efficient introduction of precise knock-ins into the human genome remains a challenging problem. DSB formation outcomes depend on the complex interaction of the GE and intracellular machinery with the range of variable parameters beginning with characteristics of different cell types, choice of transfection method and ending with different propensity of clinically relevant loci for insertion, hampering the development of a robust, universal and reproducible method. The results of published experiments clearly demonstrate the key role of the type and characteristics of templates in HDR efficiency with no “magic bullet” among a range of the proposed DNA donor’s configurations. The basic choice is currently between viral and non-viral donor templates. Evolutionary adapted for introduction of exogenous genetic material, viral vectors provide both intracellular and nuclear transport with transgene protection, remaining a mainstay for preclinical development of HSPC-based gene therapy products to date. As experience with viral vectors increases, their drawbacks, such as complex and extremely costly production as well as toxicity, which is especially important for *in vivo* setting, are being recognized as the challenges for clinical translation limiting the practical adoption of gene therapies. For wide application of genome editing, the use of synthetic templates might offer a more flexible and scalable solution. Therefore, non-viral donor templates are becoming increasingly relevant due to potential for robust and affordable manufacturing of these vectors on a clinical scale. The correct approach to the choice of donor template among variety of types proposed to date should be further based on the particular tasks and characteristics of gene therapy product. The specifics of the culturing protocol and transfection method should be taken into account, as well as selected editing tool. Finally, the donor template sequence and structure optimization should be performed since it is one of the defining parameters that determine insertion pathway, its efficiency and the success of gene therapy. Systematic studies comparing design elements of synthetic templates are still lacking, defining the need for HDR protocol tuning in each case, and highly demanded in this rapidly developing research field.

## Footnotes

1
https://www.cgtlive.com/view/upstaza-approved-aadc-deficiency-uk

2
https://eu.idtdna.com/pages/tools/alt-r-crispr-hdr-design-tool

3
https://gt-scan.csiro.au/cune
